# The Three-Level Model of Factors Contributing to High-Intensity Intermittent Performance in Male Soccer Players

**DOI:** 10.3390/ijerph192416402

**Published:** 2022-12-07

**Authors:** Juraj Pecho, Zuzana Kováčiková, Ľuboslav Šiška, Martin Mikulič, Marcel Čurgali, Lovro Štefan, Erika Zemková

**Affiliations:** 1 JP Sports—Athletes Performance Assessment, 810 00 Bratislava, Slovakia; 2 Institute of Physical Education and Sport, Pavol Jozef Šafárik University, 040 11 Košice, Slovakia; 3 Department of Physical Education and Sports, Faculty of Education, Catholic University, 034 01 Ružomberok, Slovakia; 4 Department of Sports Games, Faculty of Physical Education and Sport, Comenius University, 814 69 Bratislava, Slovakia; 5 Department of General and Applied Kinesiology, Faculty of Kinesiology, University of Zagreb, 10 000 Zagreb, Croatia; 6 Department of Sport Motorics and Methodology in Kinathropology, Faculty of Sports Studies, Masaryk University, 625 00 Brno, Czech Republic; 7 Department of Recruitment and Examination (RECETOX), Faculty of Science, Masaryk University, 625 00 Brno, Czech Republic; 8 Department of Biological and Medical Sciences, Faculty of Physical Education and Sport, Comenius University, 814 69 Bratislava, Slovakia

**Keywords:** yo-yo intermittent recovery level 2 test, aerobic abilities, anaerobic abilities, field tests

## Abstract

High-intensity intermittent performance in soccer is widely assessed using the yo-yo intermittent recovery level 2 test (YYIR2). This test is usually associated with aerobic–anaerobic performance. However, less is known about the direct or indirect contributions of abilities, including the anaerobic component. This study aims to propose a three-level model of factors contributing to YYIR2 performance, based on the investigation of relationships with aerobic endurance, repeated-sprint ability (RSA), and the linear and change-of-direction speed and power variables. Eighteen soccer players performed the YYIR2, with a 20-m shuttle run test (20mSR), an RSA test with change-of-direction, 5-m and 20-m sprints, and a 505 test, countermovement jump, squat jump, and drop jump. The results showed a significant relationship between the YYIR2 distance and the 20mSR distance (*r* = 0.721, *p* = 0.001), as well as with the RSA test mean time (*r* = −0.594, *p* = 0.009). In the second level, the 20mSR distance performance was not associated with any of the speed and power variables. However, the RSA test mean time correlated with the 5-m sprint (*r* = 0.587, *p* = 0.010), 20-m sprint (*r* = 0.702, *p* = 0.001), and 505 test (*r* = 0.585 *p* = 0.011) performance. In the third level, the 20-m sprint time was related to the squat jump (*r* = −0.577 *p* = 0.012) and countermovement jump (*r* = −0.768 *p* < 0.001) heights. In addition to aerobic endurance, this study highlights the importance of the anaerobic component in YYIR2 performance. More specifically, aerobic endurance (52%) and RSA (36%) are the main determinants of YYIR2 performance. Subsequently, the RSA performance is determined by the linear (34–49%) and change-of-direction speed (35%), while the explosive power of lower limbs contributes to sprinting performance (33–59%). Coaches should focus on the development of these abilities to improve the high-intensity intermittent performance of soccer players.

## 1. Introduction

Soccer is an intermittent sport characterized by repeated high-intensity actions, interspersed with periods of lower intensity or submaximal work. During a 90-min match, players attain 80–90% of their maximal heart rate [1], which corresponds to an oxygen uptake of around 70–80% of VO_2max_. This implies that the aerobic energy system is the dominant energy pathway during the soccer game. Conversely, the elite players’ performance is characterized by approximately 1350 activities during a game, with a change in activity every 4–6 s [2]. Many of these activities include short, intense actions, such as maximal sprints, accelerations, decelerations, changes in running direction, jumps, tackles, and ball contacts [3,4,5]. Therefore, from a physiological perspective, soccer is a sport making a considerable demand on the players’ aerobic and anaerobic energy systems [1,2].

Nowadays, soccer training is approached much more systematically than in the past, while the importance of a quantitative analysis of the game has also increased [6]. This type of analysis gives coaches and players a way to gain solid information to optimize their training.

Performance testing is an integral part of soccer training. Using valid and reliable tests that reflect real on-field performance is essential for tracking changes and developing training protocols for enhancing individual and team performance over time.

The ability to perform repeated short-term high-intensity activities throughout the match is considered a crucial element of the physical performance of soccer players and needs to be tested [5]. The yo-yo intermittent recovery level 2 test (YYIR2) is a widely used field test in soccer that assesses a player’s repeated high-intensity performance, which has a high aerobic–anaerobic energy contribution [7].

Given that the YYIR2 covers high-intensity runs, accelerations, decelerations, and turns, thereby stimulating the aerobic and anaerobic energy systems [7], it could be useful to investigate the association between all these abilities and test performance. Studies are still needed regarding this type of investigation in male soccer players. For instance, previous research evaluated the relationships between YYIR2 performance and repeated-sprint ability (RSA) in 7 × 35 m (13,14), 7 × 30 m [8], and linear speed performance in 10-, 20-, and 35-m sprints (14). These authors did not use the same distances as are covered in the YYIR2. Moreover, while the association between the YYIR2 and change-of-direction speed has been evaluated in female soccer players [9], no study has evaluated this association in male players.

This study is innovative, as it: (1) evaluates the association between performance in the YYIR2 and performance in tests of aerobic endurance, repeated-sprint ability, linear and change-of-direction speed, and jump performance; (2) uses the same running distances as covered in the YYIR2; (3) uses change-of-direction with the same angle of the turn as is used in the YYIR2. There is no other study of soccer dealing so comprehensively with performance in the YYIR2, in relation to other abilities. The study aims to propose a three-level model of factors contributing to YYIR2 performance. It will provide insights to help strength and conditioning coaches to design training programs to improve the high-intensity intermittent performance of soccer players. These findings could also benefit players of other sports where the ability to perform intense intermittent exercise is important.

## 2. Materials and Methods

### 2.1. Experimental Approach to the Problem

**Figure 1:**
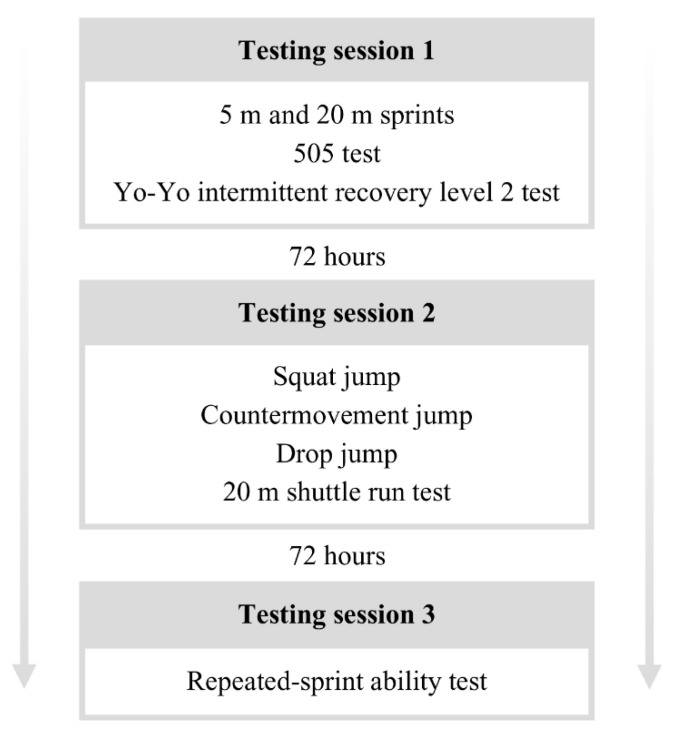
Testing protocol.

A cross-sectional correlation analysis study was used to investigate the association between performance in the YYIR2 and performance in other field tests in soccer players. Participants were tested at the beginning of the preseason for three days, with 72 h of non-testing between each session (Figure 1). All tests were performed indoors and at the same time of day. A 15-min warm-up consisting of low-intensity jogging, dynamic stretching, running drills, and task-specific high-intensity activity was completed before the tests. The participants were allowed to perform low-intensity physical activity between the testing days.

### 2.2. Subjects

A total of 18 male soccer players (age, 21.8 ± 3.1 years; height, 178.6 ± 5.8 cm; body mass, 75.2 ± 6.1 kg), consisting of 7 defenders, 9 midfielders, and 2 forwards, participated in this study. The players were from the third-best league Slovakian soccer team. They were free from orthopedic and neurological injuries. This study was approved by the Institutional Ethics Committee. The procedures that are presented were in accordance with the ethical standards on human experimentation stating incompliance with the Helsinki Declaration. All subjects were informed of potential risks and signed a written informed consent before data collection.

### 2.3. Procedures

#### Yo-Yo Intermittent Recovery Level 2 Test

The test consisted of repeated 2 × 20-m shuttle runs at a progressively increased speed, controlled by audio bleeps from a CD player. Between each shuttle, the players had a 10-s active rest period, consisting of 2 × 5-m periods of jogging [10]. Players were required to complete as many shuttle runs as possible. The test was considered to have ended when the player was unable to follow the specific pace for 2 successive shuttles or he stopped because of exhaustion. The total distance covered was recorded.

### 2.4. Endurance Performance

Aerobic endurance was assessed with a 20-m shuttle run test (20mSR). According to Ramsbottom et al. [11], the test consisted of repeated 20-m shuttles, performed at increasing speeds, until exhaustion. The audio cues were recorded on a CD. Players were required to complete as many shuttle runs as possible. The test was considered to have ended when the player was unable to follow the specific pace for 2 successive shuttles or when he stopped because of exhaustion. The total distance covered was recorded.

### 2.5. Repeated-Sprint Ability Performance

The protocol used for the RSA test was the same as that described by Rampinini et al. [12]. Players performed six 40-m (20 + 20 m) shuttle sprints with 180° turns, separated by 20 s of passive recovery. Participants were instructed to complete all sprints as fast as possible. Times were recorded using dual-beam photoelectric cells (Witty System, Microgate, Bolzano, Italy). The mean sprint time was calculated.

### 2.6. Speed Performance

Linear speed was assessed with 5-m and 20-m sprints. Players performed two trials of 20-m sprints with 5-m split times. All sprints were completed from a standing start, 0.5 m behind a starting line. Players were instructed to complete all sprints as fast as possible. The fastest time for both the 5-m and 20-m sprints was recorded.

Change-of-direction speed was assessed with the 505 agility test, using the protocol outlined by Draper and Lancaster [13]. Players performed a sprint for 15 m through the timing gates placed at 10 m, made a 180° turn on their preferred foot, and sprinted back for 5 m through the timing gates. The fastest time from two trials was recorded. Dual-beam photoelectric cells (Witty System, Microgate, Bolzano, Italy) were used to measure the sprint times in both tests.

### 2.7. Jump Performance

Players performed three types of vertical jumps, keeping their hands on their hips. Jump height in centimeters was estimated using the photoelectric cells system (Optogait, Microgate, Bolzano, Italy). First, they performed a squat jump, starting from a standing position, bending the knees to 90°, stopping for 3 s, and then jumping as high as possible. They were instructed to avoid any countermovement. Players then performed a countermovement jump from a standing position and were asked to jump as high as possible, immediately after rapid countermovement to a self-selected depth. During the drop jump, players step off from a 30-cm box and performed a maximal jump immediately after landing on the floor. They were instructed to jump as high and as fast as they could, with minimum ground contact time. The reactive strength index (RSI) was recorded and calculated as the jump height, divided by the contact time [14]. Subjects were instructed to keep their legs straight throughout the flight phase of the jumps. Two trials were carried out for each type of jump and the best result (using the jump height for the squat jump and countermovement jump, and the RSI for the drop jump) was taken.

### 2.8. Statistical Analyses

The data are reported as means ± standard deviation (SD). The Shapiro–Wilk test for normality was performed on all variables. Pearson’s correlation (r) and coefficient of determination (r^2^) were used to determine the relationships between the variables of individual tests. The magnitude of correlation coefficients was considered, according to Hopkins et al. [15], as being small (0.1 to 0.29), moderate (0.3 to 0.49), large (0.5 to 0.69), very large (0.7 to 0.89), and extremely large (0.9 to 1). The significance level was set at *p* < 0.05. Data analysis was performed using the statistical program, SPSS for Windows, version 18.0 (SPSS, Inc., Chicago, IL, USA).

## 3. Results

**Figure 2:**
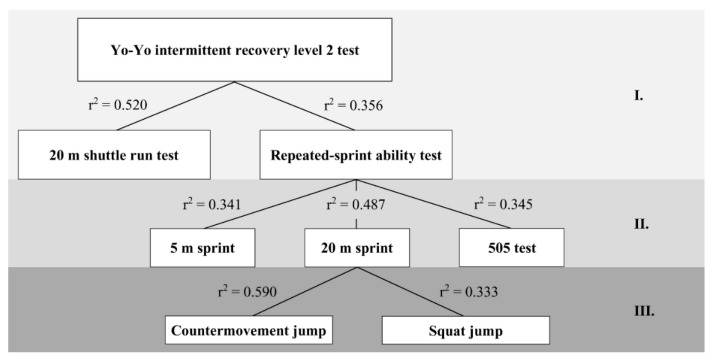
The three-level model of factors contributing to yo-yo intermittent recovery level 2 test performance. Note: r^2^ = coefficient of determination.

The individual test results are presented in Table 1. There was a large to very large relationship between the YYIR2 distance and the 20mSR distance, as well as with the RSA test mean time. Other relationships between the YYIR2 distance and the variables of speed and jump tests were not significant. While the 20mSR distance was not associated with any of the speed and jump test variables, the RSA mean time was correlated largely to very largely with the 5-m sprint, 20-m sprint, and 505 test times. In addition, the 20-m sprint time was correlated largely to very largely with the countermovement jump and squat jump heights. The all-correlation data are shown in Table 2 and the coefficients of determination are depicted in Figure 2.

## 4. Discussion

We evaluated the association between performance in the YYIR2 and performance in other frequently used field tests assessing aerobic and anaerobic abilities in soccer players. The main finding of our study is that performance in the YYIR2 was related to aerobic endurance and repeated sprint ability (RSA). Furthermore, we analyzed the associations between performance in individual field tests, with the additional aim of identifying those variables that may affect YYIR2 performance indirectly. We observed that RSA performance was associated with linear and change-of-direction speed. Additionally, linear speed was correlated with jump performance.

We found that YYIR2 performance was related to the distance covered in the 20mSR and the mean sprint time in the RSA test. These variables explained 52% and 36% of the variance of YYIR2 performance. The 20mSR assessment is generally considered a test to evaluate aerobic endurance [11]. It has been previously confirmed that the YYIR2 maximally stimulates the aerobic energy system [7]. Moreover, several studies found a significant association between YYIR2 performance and VO_2max_ in soccer players, depending on their level of performance [16,17,18]. Our findings are in line with previous studies, which observed moderate to very large associations between RSA and YYIR2 performance in soccer players [16,19]. However, they used 7 × 35-m straight-line sprint protocols, which measurement does not correspond to the YYIR2 running distances.

The RSA performance in soccer players was reported to be influenced by both aerobic and anaerobic physiological factors [20,21,22]. However, it seems that RSA performance depends more on short-sprint qualities and jump performance than aerobic fitness variables [20,21]. We found a significant association between RSA performance and the 5-m sprint, 20-m sprint and 505 test times, wherein the 20-m sprint showed the highest explained variance (49%). This indicates that the RSA 6 × 40-m test highly depends on linear sprint qualities. Several studies using 7 × 35-m [19], 10 × 20-m [23], and 7 × 30-m [8] RSA protocols also reported large to very large relationships with linear sprint performance in soccer players of different competition levels. Given that finding, speed and power should be regarded as relevant factors for RSA performance. For completeness, it should be noted that countermovement and squat jumps were significantly correlated with the 20-m sprint time. This is not surprising, as it is well known that power is associated with linear sprint performance in soccer players [24,25].

Our results confirmed the predominantly aerobic nature of the YYIR2, with a relevant anaerobic component, in terms of overall test performance. The players from our study achieved an average distance of 689 m in the YYIR2, ranging from 400 to 960 m. This is significantly shorter than for top-class players, who reach more than 1000 m on average [26]. These comparisons point to a weak level of ability to perform repeated high-intensity exercise in the players we tested. As aerobic endurance and RSA showed the closest association with performance in the YYIR2, we believe that their insufficient levels could be one of the main reasons for the weak repeated high-intensity performance. We probably see an explanation for this in the lack of aerobic high-intensity and, especially, anaerobic repeated-sprint and speed endurance training. Several studies confirm that the inclusion of these types of training increases performance in the YYIR2 in soccer players [18,27,28,29,30,31,32,33] (8,9,11,12,15,22,31,32). As RSA plays an important role in YYIR2 performance, evidence for the association of individual anaerobic abilities with RSA would help strength and conditioning coaches to design training programs that improve this ability. We observed that linear and change-of-direction speed were associated with RSA performance. Therefore, anaerobic alactic speed training, consisting of powerful accelerations and change-of-direction movements, should also be incorporated into training sessions in order to improve the high-intensity intermittent performance of soccer players. Moreover, the inclusion of exercises that improve the lower limbs’ explosive power and capacity to use elastic energy would be beneficial in the development of linear and COD speed when training soccer players.

To our knowledge, this is the first study evaluating the relationship between performance in the YYIR2 and performance in field tests of aerobic endurance, repeated-sprint ability, linear speed, change-of-direction speed, and jump performance in male soccer players. The aim was to propose a three-level model of the factors contributing to YYIR2 performance. The obtained results provide a picture of the integral character of YYIR2 performance and its gradual construction. We conducted research on soccer players from the third-best Slovakian soccer league. Therefore, further studies aimed at creating a similar model of the factors contributing to YYIR2 performance, carried out on top-class players, would be beneficial.

## 5. Conclusions

We have designed a three-level model of factors contributing to high-intensity intermittent performance in soccer players, as represented by the yo-yo intermittent recovery level 2 test (YYIR2). Aerobic endurance (52%) and repeated-sprint ability (36%) are the main determinants of YYIR2 performance. Subsequently, repeated-sprint ability performance is determined by the linear (34–49%) and change-of-direction speeds (35%), while aerobic endurance performance is not associated with speed variables or with lower-limb explosive power. Finally, the explosive power of the lower limbs contributes to 20-m sprint performance (33–59%). Identifying factors that, to varying extents, determine YYIR2 performance may assist coaches in developing training strategies to maximize improvement of the high-intensity intermittent capabilities of soccer players.

## Figures and Tables

**Table 1:** The performance test results.

Test	Mean ± SD
YYIR2 (m)	689 ± 153
20-m shuttle run test (m)	2184 ± 338
Repeated sprint ability test (s)	7.29 ± 0.18
5-m sprint (s)	1.00 ± 0.05
20-m sprint (s)	2.99 ± 0.07
505 test (s)	2.33 ± 0.08
Countermovement jump (cm)	40.5 ± 5.4
Squat jump (cm)	34.2 ± 4.2
Drop jump (RSI)	1.93 ± 0.31

Note: YYIR2 = Yo-yo intermittent recovery level 2 test; RSI = reactive strength index.

**Table 2:** The Pearson’s r correlation analysis results.

**Level 1**									
		20mSR	RSA test	5 m	20 m	505 test	SJ	CMJ	DJ
YYIR2	*r*	**0.721**	**−0.594**	−0.233	−0.457	−0.343	0.249	0.332	0.071
	*p*	**0.001**	**0.009**	0.351	0.057	0.163	0.320	0.178	0.780
**Level 2**									
		5 m	20 m	505 test	SJ	CMJ	DJ		
RSA test	*r*	**0.587**	**0.702**	**0.585**	−0.185	−0.452	−0.123		
	*p*	**0.010**	**0.001**	**0.011**	0.462	0.060	0.627		
**Level 3**									
		SJ	CMJ	DJ					
20 m	*r*	**−0.577**	**−0.768**	0.092					
	*p*	**0.012**	**<0.001**	0.718					

Note: statistically significant associations are highlighted in bold; YYIR2 = yo-yo intermittent recovery level 2 test; 20mSR = 20-m shuttle run test; RSA = repeated sprint ability; CMJ = countermovement jump; SJ = squat jump; DJ = drop jump.

## Data Availability

The data presented in this study are available on request from the corresponding author.
